# Brazil’s Path to Vaccine Recovery and Leadership

**DOI:** 10.1590/0037-8682-0308-2025

**Published:** 2025-12-19

**Authors:** Ethel Leonor Maciel, Eder Gatti Fernandes, Guilherme Loureiro Werneck, Paulo Eduardo Guedes Sellera, Ana Estela Haddad, Nisia Trindade Lima

**Affiliations:** 1 Universidade Federal do Espírito Santo, Vitória, ES, Brasil.; 2 Programa Nacional de Imunizações, Ministério da Saúde, Secretaria de Vigilância em Saúde e Ambiente, Brasília, DF, Brasil.; 3 Secretaria Vigilância em Saúde e Ambiente, Ministério da Saúde, Brasília, DF, Brasil.; 4 Instituto de Estudos em Saúde Coletiva, Universidade Federal de Rio de Janeiro, Rio de Janeiro, RJ, Brasil.; 5 Instituto de Medicina Social, Universidade do Estado do Rio de Janeiro, Rio de Janeiro, RJ, Brasil.; 6 Secretaria de Informação e Saúde Digital, Ministério da Saúde, Brasília, DF, Brasil.; 7 Faculdade de Odontologia, Universidade de São Paulo, São Paulo, SP, Brasil.; 8 Fundação Oswaldo Cruz, Rio de Janeiro, RJ, Brasil.

In a recent editorial^1^, we described how several challenges hindered vaccination efforts. In recent years, Brazil has faced a problem in the field of immunization, marked by a steady decline in vaccination coverage and the resurgence of vaccine-preventable diseases, such as measles. Between 2016 and 2021, the national vaccination rates decreased significantly, falling below 70% for essential vaccines in some regions, contributing to illness and death from vaccine-preventable diseases in the country. In 2019, this decline led to the loss of Brazil’s measles-free certification and its inclusion in UNICEF’s list of the 10 countries with the highest number of unvaccinated children^2^ ^,^ ^3^.

However, a series of digital policies and strategies drove recovery in 2023 and 2024, marking a turning point. The implementation of evidence-based communication campaigns, along with consistent public policies aimed at increasing access to vaccines and modernizing immunization data systems, allowed for the recovery of vaccine coverage^1^. 

The first was a targeted effort involving agreements within the Tripartite Inter-Management Commission and the resumption of specific financial transfers for vaccination campaigns, totaling BRL 351 million (USD 63 million), allocated to states and municipalities^4^ ^-^ ^7^. The initiatives included implementing local planning strategies for High-Quality Vaccination Activities, designed to organize and optimize vaccination, identify population needs, improve resource allocation, and overcome operational constraints^8^ ^,^ ^9^.

Second, the Brazilian government adopted a Vaccination Strategy Monitoring system. This approach aims to assess field coverage, identify operational barriers, optimize resources, correct deviations in real time, and ensure equitable vaccine access. A total of 1,554,030 households were visited, an unprecedented effort that resulted in a measured coverage rate of 91.6% for two doses of the measles vaccine among children under five.

Third, funding for school-based vaccinations in states and municipalities has become a central pillar in efforts to expand vaccination coverage among school-aged children and adolescents in Brazil. According to the 2023 local planning report and updated data compiled in May 2025, these initiatives continued to expand nationally. Currently, 3,710 municipalities have implemented school vaccination programs. Between 2023 and 2024, the federal government allocated BRL 150 million (USD 27 million) to support school-based vaccination strategies.

Fourth, the expansion of the Brazilian Unified Health System Digital and the integration of the National Health Data Network with the National Health System User Registry, using Brazil’s Individual Taxpayer Number as a unique identifier, enabled individual tracking and real-time vaccine surveillance. This open and qualified information enhances trust, broadens National Immunization Program (NIP) transparency, and strengthens the Brazilian healthcare system.

Fifth, the impact of misinformation and disinformation on vaccination coverage was assessed. To this end, the launch of the Health with Science program was crucial in combating fake news about vaccination^10^. The combination of these strategies has increased Brazil’s coverage. The first dose of the measles, mumps, and rubella (MMR) vaccine reached 95% coverage by 2024, meeting the target of the NIP for the first time in nearly a decade^3^ ^,^ ^4^. In November 2024, these advances led to the recertification of Brazil as free of endemic circulation of measles, rubella, and congenital rubella syndrome by the Pan American Health Organization^5^. In the same year, Brazil was officially removed from UNICEF’s list of the 10 countries with the worst childhood vaccination indicators, a milestone of both symbolic and epidemiological significance^3^.

Challenges persist, and efforts must continue. Although we previously understood that vaccination efforts, once achieved, did not require additional efforts, we now learn that challenges are ongoing, and we need continued attention to vaccination efforts. In Brazil, significant regional inequalities exist, which are reflected in vaccination coverage. 

A population-based study conducted in the Northeast region analyzing the 2017-2018 birth cohort reported that the coverage of the MMR vaccine among children up to 24 months of age was only 79.3%, with a dropout rate of 10.6%. The absence of a vaccination card was the strongest factor associated with non-vaccination (adjusted Odds ratio= 10.7), indicating shortcomings in record-keeping and individual follow-up. Other contributing factors included low household income, a high number of children per mother, and lack of maternal employment, underscoring the direct link between immunization and social determinants of health.

Recognizing these social determinants is essential to maintain homogeneous coverage and actively monitor vulnerable areas to integrate social policies into vaccination strategies. 

Furthermore, combating vaccine hesitancy requires ongoing educational initiatives, trust-based communication, stronger ties between families, and a Unified Health System. Thus, the Health with Science program, by promoting the value of science and having a strategy for disseminating truthful information, can be a powerful driver of trust, and its action must be intensified, including holding those who disseminate false information that harms individuals lives, accountable.

Finally, rebuilding the NIP to its full capacity requires sustainable investment, robust infrastructure, a valued primary care workforce, and equity-oriented epidemiological intelligence. Brazil has re-established itself as a global leader in vaccination; however, sustaining this leadership increasingly depends on our ability to ensure that no child anywhere in the country is left unprotected.

## References

[1] Fernandes EG, Werneck GL, Haddad AE, Maciel ELN, Lima NVT (2024). Restoring High Vaccine Coverage in Brazil: Successes and Challenges. Rev Soc Bras Med Trop.

[2] Domingues CMAS, Maranhão AGK, Teixeira AM, Fantinato FFS, Domingues RAS (2020). The Brazilian National Immunization Program: 46 Years of Achievements and Challenges. Cad Saúde Pública.

[3] UNICEF (2023). The State of the World’s Children 2023: For Every Child, Vaccination.

[4] Brasil. Ministério da Saúde (2023). Portaria nº 844, de 14 de julho de 2023. Dispõe sobre ações de multivacinação no âmbito do Sistema Único de Saúde - SUS para o exercício de 2023, incluindo a instituição de incentivo financeiro de custeio, excepcional e temporário, para esse fim.

[5] Brasil. Ministério da Saúde (2024). Portaria nº 3.288, de 08 de março de 2024. Estabelece incentivo financeiro de custeio, de caráter excepcional e temporário, para o desenvolvimento da Estratégia de Vacinação nas Escolas, da Campanha Nacional de Vacinação contra a Poliomielite e do Monitoramento das Estratégias de Vacinação no Brasil, no âmbito do Sistema Único de Saúde - SUS, em 2024.

[6] Brasil. Presidência da República (2023). Decreto nº 11.531, de 16 de maio de 2023. Dispõe sobre convênios e contratos de repasse relativos às transferências de recursos da União, e sobre parcerias sem transferências de recursos, por meio da celebração de acordos de cooperação técnica ou de acordos de adesão.

[7] Brasil. Presidência da República (2025). Autoriza, em caráter excepcional, a prorrogação dos prazos para atendimento das cláusulas suspensivas dos convênios e contratos de repasse celebrados no período de 1º de setembro.

[8] Ministério da Saúde (2024). Cresce a cobertura de 15 das 16 vacinas do calendário infantil.

[9] Organização Pan-Americana da Saúde (OPAS) (2024). Brasil é recertificado como livre da circulação do sarampo, rubéola e síndrome da rubéola congênita.

[10] Ministério da Saúde (BR) (2025). Saúde com Ciência.

